# First person – Monica Shukla

**DOI:** 10.1242/dmm.052119

**Published:** 2024-10-14

## Abstract

First Person is a series of interviews with the first authors of a selection of papers published in Disease Models & Mechanisms, helping researchers promote themselves alongside their papers. Monica Shukla is first author on ‘ Neuromuscular junction dysfunction in Lafora disease’, published in DMM. Monica is a PhD scholar in the lab of Professor S. Ganesh at the Indian Institute of Technology, Kanpur, India, investigating the influence of aberrant glycogen accumulation in neurodegenerative disorders, emphasising its effects on both the brain and muscles, and exploring how mitigating glycogen levels could alleviate the associated disease phenotype.


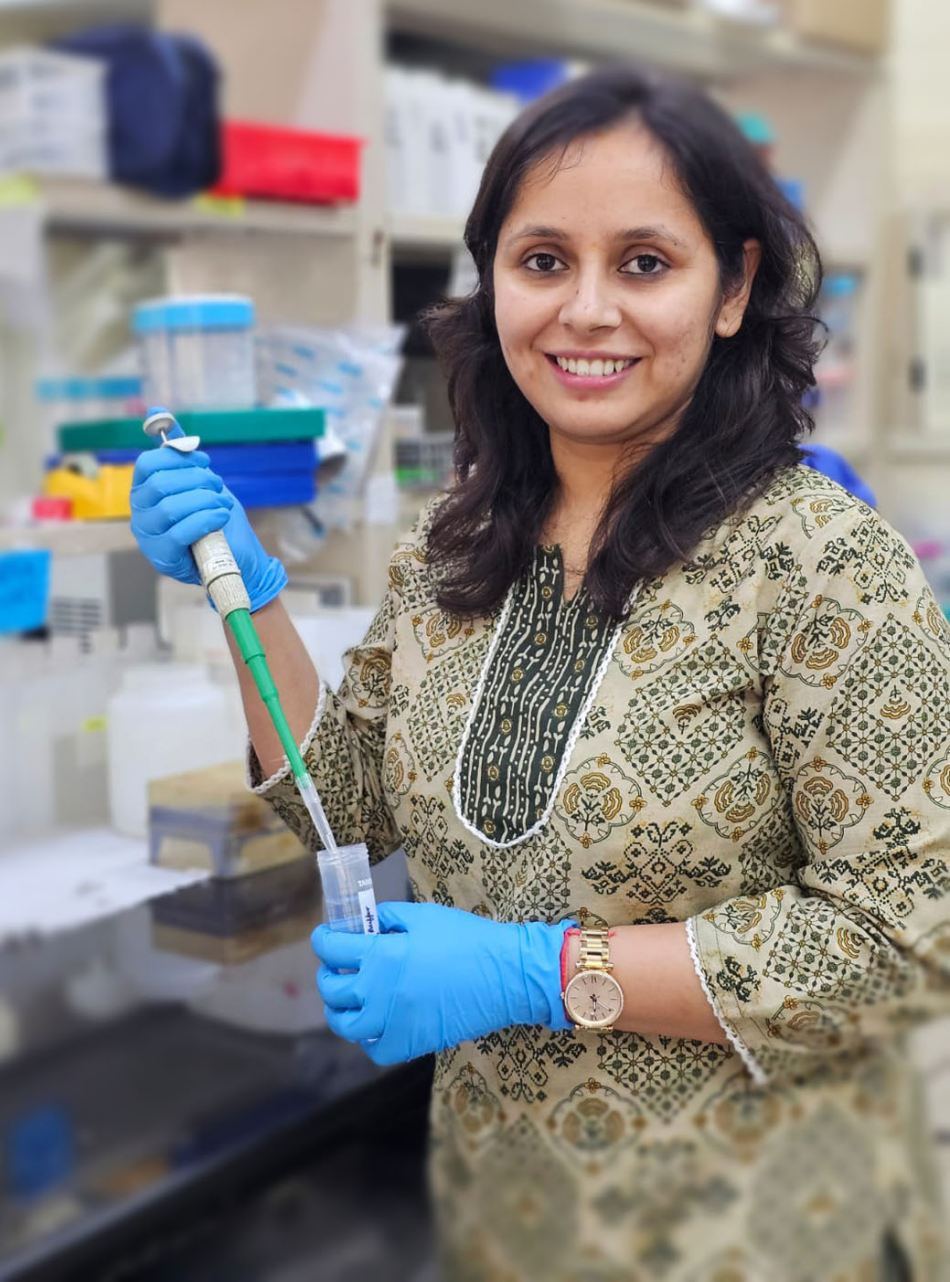


**Monica Shukla**

**Who or what inspired you to become a scientist?**

My journey into science began with a profound fascination with the human body and its extraordinary complexity. From a young age, I was intrigued by how such an intricate system could function so seamlessly and, more importantly, how it could adapt when faced with illness or injury. The body's ability to compensate for disruptions in its natural processes, through mechanisms I found both mysterious and inspiring, became the focus of my curiosity. As I delved deeper into biology, I became captivated by the balance between health and disease – how, even in the face of malfunction, our systems deploy remarkable strategies to restore equilibrium. This drive to understand the underlying mechanisms of human biology became a defining aspect of my scientific pursuits. I realised that every disease unveils a new layer of complexity, where the body's response can sometimes be as fascinating as the disease itself. The sheer intricacy of molecular pathways, cellular communication and tissue repair processes compelled me to study how the body attempts to fight, adapt or compensate for such challenges. This dedication has shaped my research and reinforced my desire to contribute to the greater understanding of human health and disease.

[…] every disease unveils a newl layer of complexity, where the body’s response can sometimes be as fascinating as the disease itself.

**What is the main question or challenge in disease biology you are addressing in this paper? How did you go about investigating your question or challenge?**

The main question I am addressing in this paper is how dysfunctions in glycogen metabolism contribute to neuromuscular junction (NMJ) abnormalities in Lafora disease (LD), a rare form of progressive myoclonus epilepsy classified as a glycogen storage disorder (GSD). While glycogen accumulation in the nervous system is a well-known feature of the disease and has been widely studied, there remains a gap in understanding the neurobiological mechanisms behind the muscle impairments experienced by LD patients. One potential mechanism involves the disruption of NMJ transmission due to aberrant glycogen accumulation, which may play a critical role in muscle dysfunction. My goal in this study was to uncover the molecular connections between glycogen metabolism and the structural and functional impairments at the NMJ, thereby advancing our understanding of how metabolic dysfunction can drive progressive neurodegeneration and muscle impairment. To investigate this, I utilised mouse models of LD wherein I used a combination of electrophysiological and molecular approaches to assess NMJ transmission and its structural integrity. Advanced microscopy techniques were used to visualise NMJ morphology and to explore the potential compensatory mechanisms within the neuromuscular system to determine whether the body attempts to counteract these disruptions. This multifaceted approach enabled me to investigate both the structural and functional consequences of glycogen accumulation, bringing us closer to understanding the metabolic basis of NMJ dysfunction in LD.

**How would you explain the main findings of your paper to non-scientific family and friends?**

Glycogen is a stored form of sugar in the body, primarily found in the liver and muscles, where it plays a vital role in providing energy for various functions. However, excessive glycogen accumulation resulting from disruptions in its metabolic pathway can lead to serious health complications, affecting tissues such as the brain, liver and muscles, and giving rise to a range of conditions classified as GSDs. One particularly severe condition linked to abnormal glycogen buildup is LD, a rare and progressive neurodegenerative disorder that typically emerges in adulthood. LD is marked by symptoms such as seizures, cognitive decline, dementia, ataxia and respiratory insufficiency – one of the leading causes of death in LD patients. While research has shed light on the mechanisms behind the neurological symptoms of LD, the effects of glycogen accumulation on muscle function remain underexplored. To address this gap, my current work examines the structural integrity and functionality of the NMJ – the critical site where nerves communicate with muscles to control movement. Through a genetic mouse model of LD, I found that NMJ transmission is compromised, impairing the ability of muscles to receive and respond to signals from the brain. This disruption in brain–muscle communication leads to functional deficits. Gaining insight into these changes is key to understanding how glycogen buildup affects muscle function and could pave the way for new treatment strategies.

**What are the potential implications of these results for disease biology and the possible impact on patients?**

The findings from my study on the NMJ in LD could have several important implications for both disease biology and potential patient outcomes. By demonstrating that abnormal glycogen accumulation disrupts NMJ transmission, impairing muscle responsiveness to neural signals in LD, this research sheds new light on how muscle functionality is compromised in this neurodegenerative disorder. The key observations – like altered electrophysiological properties, including reduced muscle output in response to repetitive nerve stimulation, reduced end-plate area and its complexity, and increased frequency of fragmented junctions – confirm the disruption in NMJ transmission in the LD mouse models. These findings expand our understanding of LD beyond its well-known neurological effects, highlighting a direct impact on muscle communication and movement control. For patients, this insight opens up new avenues for treatment strategies. If we can better understand the mechanisms by which glycogen buildup interferes with NMJ functionality, we can potentially target these pathways to mitigate motor impairments, improve quality of life, and delay or alleviate motor symptoms in LD patients. In the broader context of GSDs, this research might also inform approaches to other known GSDs, where abnormal glycogen accumulation affects neuromuscular function, potentially benefiting a wider range of patients.

DMM’s broad readership, particularly among scientists studying disease models, ensures that my findings will reach the right audience, enhancing both its visibility and potential influence in the field.

**Figure:**
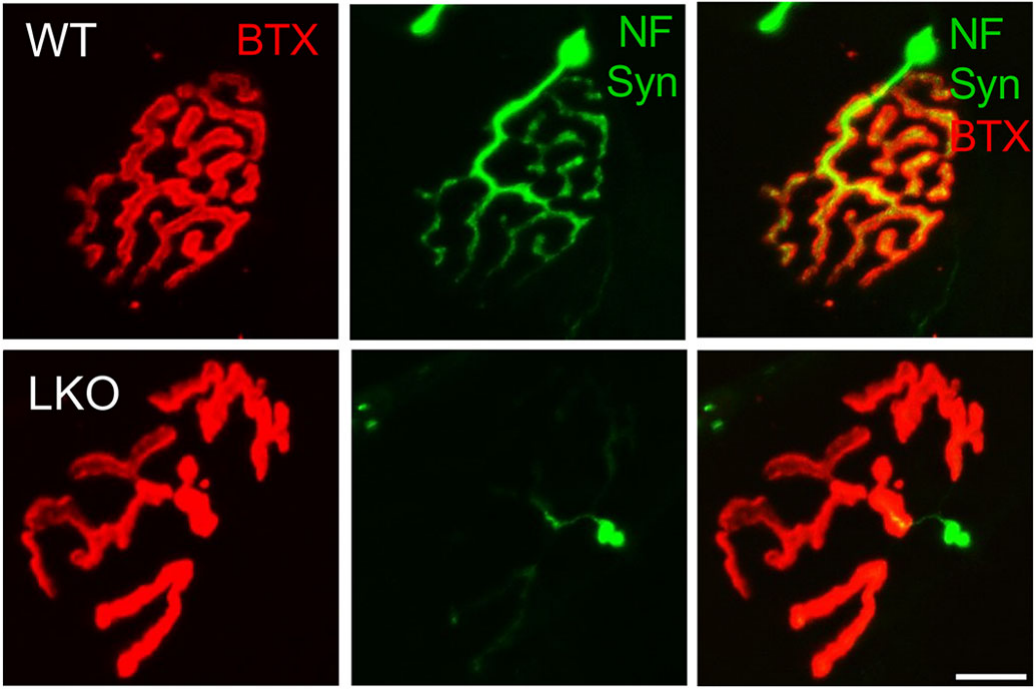
**Representative images showing the altered neuromuscular junction morphology in the gastrocnemius muscle of the Lafora disease mouse model.** BTX, α-bungarotoxin; LKO, laforin-deficient; NF, neurofilament; Syn, synapsin-1; WT, wild-type. Scale bar: 10 μm.

**Why did you choose DMM for your paper?**

I selected DMM for my paper because it is a well-respected journal known for publishing research focused on understanding human diseases through the use of model organisms and systems. Since my study explores NMJ dysfunction in LD by using a genetic mouse model, DMM offers the most suitable platform for sharing this work, which connects fundamental research to its possible applications in treating human disease. The journal's focus on disease mechanisms aligns closely with the objectives of my research, which aims to reveal how glycogen buildup affects NMJ transmission and muscle function in LD. Additionally, DMM's broad readership, particularly among scientists studying disease models, ensures that my findings will reach the right audience, enhancing both its visibility and potential influence in the field. Another key reason for choosing DMM is its open-access policy, which ensures that my work will be freely accessible to the global scientific community, including clinicians, and those working on therapeutic strategies. This open-access availability maximises the visibility of my work and enhances its potential impact across various fields.

**Given your current role, what challenges do you face and what changes could improve the professional lives of other scientists in this role?**

Enhancing the professional lives of scientists involves addressing several critical aspects. Foremost among these is the need for greater job security, especially for early-career researchers, to ensure a stable environment that supports sustained and impactful work. Additionally, increasing flexibility for researchers to engage in discussions and share ideas with a global audience would stimulate greater collaboration and innovation. Presently, a considerable amount of scientists' time is dedicated to securing research funding. Simplifying this process by establishing a central body that provides accessible funding for qualified individuals could significantly reduce administrative burdens, allowing scientists to concentrate more on their research and improve their work environment. Implementing these changes would foster a more dynamic, collaborative and supportive professional setting, ultimately advancing scientific innovation and progress.

**What's next for you?**

Looking ahead, my primary focus will be on advancing a new project that involves a novel inhibitor aimed at reducing the abnormal glycogen burden associated with various GSDs. The next phase will involve testing this inhibitor to evaluate its effectiveness in lowering glycogen levels and assessing its impact on the NMJ functionality in LD mouse models. This research aims to determine whether the NMJ dysfunction observed in these models is directly caused by abnormal glycogen accumulation or results from disruptions in other interconnected functional pathways. By elucidating these mechanisms, I hope to enhance our understanding of NMJ impairment and develop more targeted therapeutic strategies to improve muscle function in GSDs, including LD. Additionally, I am enthusiastic about the potential for this work to facilitate collaborations and drive innovative solutions that could significantly benefit patient care and treatment outcomes.

**Tell us something interesting about yourself that wouldn't be on your CV**

Something interesting about myself that wouldn't be on my CV is my deep fascination with the complexity of human biology, which goes beyond the lab. I'm particularly inspired by how the body's natural systems attempt to adapt and compensate in the face of disease. While my research focuses on the molecular mechanisms of glycogen metabolism and neuromuscular dysfunction, I'm driven by the potential to translate these findings into therapeutic strategies that could improve patient outcomes. The idea that my work could one day contribute to alleviating disease burden is what motivates me the most. In addition to my academic pursuits, I'm passionate about bridging the gap between bench and bedside – ensuring that the time and effort I put into my research can ultimately benefit those in need. This translational approach keeps me grounded in the real-world applications of my work and constantly challenges me to think about the broader implications for patient care. It's this blend of scientific inquiry and clinical relevance that makes my research journey particularly fulfilling.
